# Polydopamine-Assisted Surface Modification for Bone Biosubstitutes

**DOI:** 10.1155/2016/2389895

**Published:** 2016-08-09

**Authors:** Shishu Huang, Nuanyi Liang, Yang Hu, Xin Zhou, Noureddine Abidi

**Affiliations:** ^1^Department of Orthopedic Surgery, West China Hospital, Sichuan University, Chengdu, Sichuan 610041, China; ^2^Centre for Human Tissues and Organs Degeneration and Shenzhen Key Laboratory of Marine Biomedical Materials, Shenzhen Institutes of Advanced Technology, Chinese Academy of Sciences, Shenzhen, Guangdong 518055, China; ^3^Fiber and Biopolymer Research Institute, Department of Plant and Soil Science, Texas Tech University, Lubbock, TX 79409, USA

## Abstract

Polydopamine (PDA) prepared in the form of a layer of polymerized dopamine (DA) in a weak alkaline solution has been used as a versatile biomimetic surface modifier as well as a broadly used immobilizing macromolecule. This review mainly discusses the progress of biomaterial surface modification inspired by the participation of PDA in bone tissue engineering. A comparison between PDA-assisted coating techniques and traditional surface modification applied to bone tissue engineering is first presented. Secondly, the chemical composition and the underlying formation mechanism of PDA coating layer as a unique surface modifier are interpreted and discussed. Furthermore, several typical examples are provided to evidence the importance of PDA-assisted coating techniques in the construction of bone biosubstitutes and the improvement of material biocompatibility. Nowadays, the application of PDA as a superior surface modifier in multifunctional biomaterials is drawing tremendous interests in bone tissue scaffolds to promote the osteointegration for bone regeneration.

## 1. Introduction

Tissue engineering aims to engineer biosubstitutes to repair and regenerate injured tissues to improve the quality of life of individuals. The key to successfully fabricate such biosubstitutes requires the optimal combination of isolated cells, tunable factors, and supportive scaffolds [1–3]. Due to the complexity of tissue regeneration regarding various cases of malformations, occasional accidents in regeneration, chronic infections, and functional failure of end-organs, the biosubstitute prototyping becomes extremely intricate and individualized [4–7]. Musculoskeletal diseases are facing challenges generated by the rapid increase of worldwide aging problem, most of which need the assistance of foreign substitutes to heal during common clinic orthopedic operations such as bone filling and articular replacement [7]. Living tissue donation is considered the best choice for most cases regardless of some implants that may have the nonnegligible drawbacks such as allogeneic diseases, foreign body reactions, and immunogenicity [8–10]. However, due to increasing demand and shortage of living tissue sources, the artificial biosubstitutes become a promising alternative. Nowadays, biosubstitute has benefited from different materials and processing technologies, such as decellularized matrix, biomimetic scaffolds, and multidimensional organ printing [11]. For example, in order to prevent inflammation, the use of cationic Zn with high concentration has been incorporated into biomaterials as an anti-inflammatory molecule to trigger immune cells (polymorphonuclear cells, PMNs) in acute responses [12–14]. A vessel network was used to construct thick 3D bone implants in order to improve vascularization by integrating microfabrication, signaling cell coculturing, and prolonging the release of angiogenesis growth factors during the regeneration of injured bone tissues [15, 16].

The surface modification of bone biosubstitutes is an advanced technology that is capable of awarding biosubstitutes multiple functions to better meet the requirements of bone regeneration, such as cell affinity, material biocompatibility, nontoxicity, and biodegradability. Approaches to traditional surface modification are usually limited by tedious preparation steps and rigorous reaction conditions, which are thereby restrained to limited number of material categories [17]. Comparably, surface modification inspired by polymerized dopamine (polydopamine, PDA) has recently become a simple, safe, effective, and cost-saving alternative way to satisfy the requirements of bone tissue engineering. PDA-assisted surface modification is strongly subjected to the formation of PDA coating on the biomaterial interface caused by the oxidation reaction of dopamine (DA) in weak alkaline solution. PDA coating exhibits extensive chemical properties spanning from the protection against light damage to the role as biosensors transmitting the biological information, among which the adhesive function of PDA coating is one of the most useful features associated with bone tissue regeneration [18]. It can act as a modifier on the interface of different bone biosubstrates and improve their interfacial properties that may provide considerable options for tissue constructs.

## 2. PDA and the Formation Mechanism of PDA Coating Layer

The adhesive function of polydopamine originates from the study of mussel adhesive proteins (MAPs) secreted by marine mussel (e.g.,* Mytilus edulis*). It was found that MAP is able to form adhesive “feet” to attach to different materials in humid environment and even rigorously trembling environment [19]. MAPs contain abundant 3,4-dihydroxy-L-phenylalanine (DOPA) that enable mussel organism to adhere to almost all kinds of materials via covalent and noncovalent bonding [19, 20]. As a precursor of PDA, DA has functional groups (catechol and phenethylamine) and adhesive mechanism similar to DOPA, which offers DA the potential as a versatile surface modifier in various biomedical applications [21–23]. DA was first applied as an important hormone and neurotransmitter of central nervous system in the treatment of Parkinson's disease in the early 1970s [24]. It plays an essential role in acquisition, emotional regulation, drug addiction, and behavioral control [25–28]. The bioadhesive application of DA has been documented, which highlights PDA for the same adhesive purpose because PDA is derived from DA and it has characteristics similar to DA [29]. In addition, due to the composition similar to some proteins in organism, PDA can also offer some bioprotection functions, such as photoprotection, reactive oxygen species scavenging (ROS), and metal cation sequestering [30, 31].

**Figure 1:**
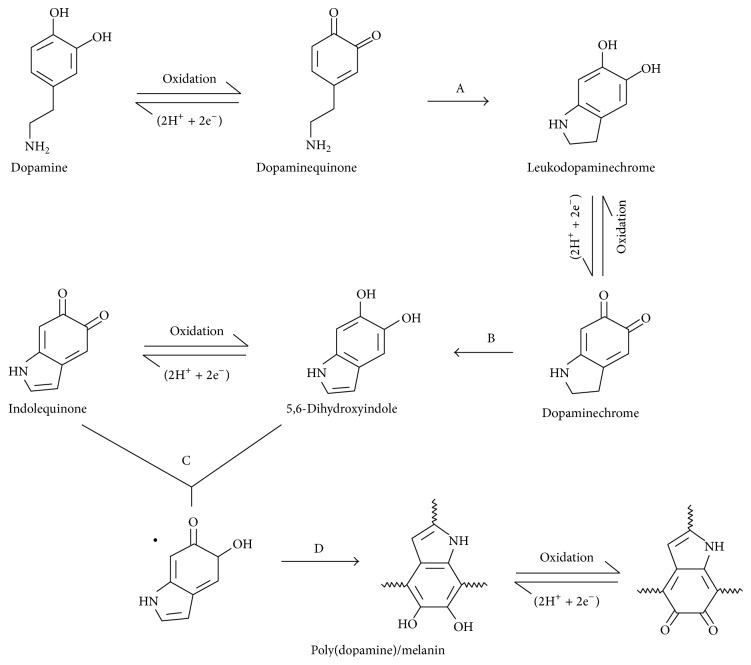
The schematic formation of PDA coating layer [31].

**Figure 2:**
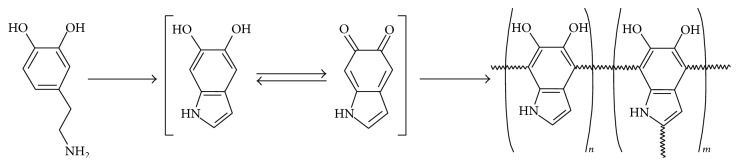
Different monomer states during the polymerization of DA to form PDA layer [39].

To date, the adhesive function of PDA enables it to become a useful biomimetic surface modifier and biomolecule immobilizer. However, the adhesive mechanism of PDA on the interface of various substrates still remains debating due to the complexities of structural analysis of PDA and its analogous molecules such as eumelanin [32–35]. The analogy of PDA to eumelanin is a useful approach to understand the chemical formation of PDA layer [36]. Eumelanin is naturally formed with the L-3,4-dihydroxyphenylalanine (L-DOAP) or tyrosine as precursors. These DA based monomers and other important conditions of PDA polymerization become decisive factors to impact the formation of PDA layer [31, 37]. Several mechanisms of the PDA coating layer have been developed and they all consist essentially of two steps as shown in Figure 1, including the autooxidation and intramolecular rearrangement of DA monomers and polymerization of DA monomers [29, 31, 38]. The first step involves the occurrence of autooxidation of DA monomers where dopaminequinone is synthesized in the presence of dissolved oxygen, alkaline buffer, and adequate supply of initial dopamine monomers. Dopaminequinone is subjected to intramolecular cyclization (A substep), reversible oxidation to form dopaminechrome, and intramolecular rearrangement to lead to 5,6-dihydroxyindole (B substep). The reverse dismutation reaction occurs between 5,6-dihydroxyindole and its further oxidized intermediate (indolequinone) (C substep) finally leads to PDA (D sub-step) [31]. The homomonomer and heteromonomer of indoles in various oxidation states are shown in Figure 2 [39]. The second step of PDA formation still remains elusive. Several popular models have clearly addressed the polymerization of DA monomers. The first model exhibits a “covalent bonding” polymerization fashion where DA is polymerized via 5,6-dihydroxyindole monomers following further oxidization and subsequent intermolecular cross-linking of PDA [29, 40–44]. The second model is the “noncovalent bonding (quinhydrones)” polymerization fashion where DA monomers are linked together by noncovalent interaction including hydrogen bonding, charge transfer, ionic interaction, and *π*-stacking to aggregate into a structural macroassembly [35, 45–47]. As PDA polymer units, the polymeric synthesis of quinhydrones has been well documented by the oxidative polymerization using biomacromolecules or supramolecules as a template [48, 49]. The “noncovalent bonding” fashion is a controversial assumption that has not been broadly acknowledged while the “covalent bonding” assumption has been partly proved via C-C bonding among the benzene rings of the dihydroxyindoline and indolinedione and open-chain monomer units [38, 50].

## 3. Immobilization of Functionalized Biomolecules

Upon the occurrence of oxidative polymerization of DA, PDA coating layer can be perfectly formed on various interfaces of substrates including noble metals, oxides, ceramics, polymers, and semiconductors [29]. Such a PDA coating layer with tunable thickness of 10–100 nm as functions of incubation time and various material interfaces [51, 52] can offer good surface properties such as high hydrophilicity, long-term corrosion resistance, and moderate physicochemical properties [31, 53, 54]. In order to optimize versatile functions of PDA coating in real environments, some additional biomolecules are considered to anchor on the PDA coating layer. Nucleophilic agents containing functional groups such as amino, thiol, imidazole, and mercapto can readily attack the catechol or diketone groups exposed on the PDA layer via Shiff-base and Michael addition reactions so that to provide improved surface properties to the biosubstitutes [29, 55, 56]. In addition, biomacromolecules, such as proteins, peptides, and DNA, can anchor on the surface of biosubstitutes via the PDA coating layer [50, 57–60]. Such coordination bonding and strong chelating bonding with transition metal compound occur between catechol or diketone groups on the PDA coating layer and the interface of inorganic biosubstitutes along with the protein cross-linking [61–66]. Meanwhile, on certain specific surfaces of inorganic biosubstitutes (e.g., negatively charged oxidized silicone), the electrostatic interaction from protonated amino group can also enhance the adhesion of biomolecules to the surface of biosubstitutes [62, 67]. For the surface of organic material, the oxidation of catechol group exposed on the PDA coating layer results in the formation of quinone/semiquinone to form irreversible covalent bonding [19] between biomolecules and the surface of organic material via similar Michael addition and Schiff formation reactions [29] and thereby further trigger the intermolecular cross-linking reactions among PDA polymers. It should be noted that the DA and DOPA exhibit similar surface-modification function due to the same functional groups and similar structures. However, such a function of surface modification inspired by PDA coating layer varies according to different biosubstitutes and different immobilized biomolecules [68].

## 4. Advantages for Applications of PDA in Bone Biosubstitute

Although bone graft is commonly used in clinical treatment (such as autograft, allograft, and xenograft) it is still not ideal for treatment of bone defects. The ideal scaffold of biosubstitute materials for bone defects aims to enable cell attachment and proliferation, possess osteodifferentiation ability, and implement mineralization performance. PDA-assisted surface modification can offer these properties described in the following section.

PDA has been reported to be one of the mineral inducers to promote the formation of mineralized surface. It is known that nanohydroxyapatite (nano-HAp) and collagen are two major components of natural bone. The capability of bone biosubstitute inducing the mineralization containing the nucleation of calcium and the crystallization of nano-Hap in a real wound environment is an important index to evaluate the effectiveness of bone repair [69]. The bone biosubstitutes can be fabricated from many different material categories from metals, inorganics to organics, such as noble metals, metal oxides, ceramics, and polymers. The formation of PDA coating layer on the silicon-based biosubstitutes has been found to assist in the mineralization [23]. In addition, under the assistance of PDA coating layer, the HA coated biosubstitute surface (e.g., titanium alloy, Ti6Al4V) can immobilize some active biomolecules such as BMP-2, and these immobilized biomolecules can be uniformly distributed on the surface and exhibit a sustainable release profile once in a real bone repair environment [70].

PDA is noncytotoxic and the formed PDA coating layer can control cellular behaviors. It has been reported that PDA enhances the proliferation and calcium deposition of osteoblast cells and the effect can be further strengthened in the combination of growth factors [71, 72]. The immobilization of rabbit chondrocytes has been improved with the formation of PDA coating layer on biosubstitutes as compared to unmodified surface. Additionally, the PDA coated surface can enhance the attachment of NT3T3 fibroblast cell [73, 74]. The amine-(thiol-) terminated methoxy-poly(ethylene glycol) (mPEG-SH/mPEG-NH_2_) with PDA coating layer can improve the cell adhesion behavior although this biosubstitute material exhibits an adverse effect of inhibiting the growth of NIH 3T3 cells [29]. Meanwhile, PDA can be used as controllable surface modifier to reinforce the selective antifouling of biomaterial surface or the cell affinity of scaffold. Tsai et al. [75] gave an example of performing one-step codeposition of poly(ethyleneimine)-graft-poly(ethylene glycol) (PEI-g-PEG) and PEI-g-biotin via PDA coating layer on various substrates. This enables cells to selectively attach on such PDA modified surfaces. Investigation in the cell adhesion on the material surface via codeposition of PDA and PEI-g-galactose obtained a similar outcome where cells were attached in the form of specific micropatterning profile [76]. Furthermore, mediating various parameters, such as using different cell lines, preprocessing PDA, and culturing cells under different conditions, the formed PDA coating layer may offer tunable regulating properties. For example, modulating the incubation temperature of DA (PDA precursor) could result in the improvement of the ratio of quinone versus phenolic hydroxyl groups to better regulate the proliferation of smooth muscle cells attached on the biosubstitutes [77].

For some sensitive biomolecules that are easily deactivated because of changes of environmental conditions, the formed PDA coating layer can immobilize them on the biosubstitutes and meanwhile exert no or tiny impact on their activities. Growth factors including most sensitive biomolecules, such as bone morphogenic protein (BMP), Arg-Gly-Asp peptide (RGD), and alkaline phosphatase (ALP), are able to adhere to the surface of biosubstitutes via the formed PDA coating layer. Studies have shown that the activity of these biomolecules was not affected by the PDA coating layer itself except for the forming condition of PDA coating layer and the inherent properties of biomolecules (incubation time, concentration, molecule weight, and isoelectric point) [78]. Compared to traditional immobilization approaches, PDA-assisted immobilization exhibits a merit to significantly improve the anchoring performance of biomolecules [79].

In an* in vivo* environment, it has been found that the PDA coating layer enhances the biocompatibility of bone biosubstitutes. For a PDA-coated surface of poly-L-lactic acid (PLLA) biosubstitute, the PDA coating layer reduced the blood toxicity of CdSe quantum dots while it enhanced the immunogenicity and the biocompatibility of biosubstitute material [80]. PDA-coated substrate of biosubstitute made of nylon/cellulose/polyethersulfone composite, grafted with bovine serum albumin (BSA), can reduce the attachment of blood platelets and selectively enable BSA to anchor on the surface, which leads to the decrease of unwanted blood clotting [81]. Additionally, recent studies have shown a positive outcome of restoration and regeneration of bone tissue by means of the use of PDA-coated technology to the bone implanted materials. One reported on a collagen membrane coated with PDA on which calcitriol was absorbed [82]. The osteogenic competency of this membrane composite was examined when it was implanted into rat mandibular bone defects, suggesting that calcitriol bound to PDA-coated membrane showed strong potential in prohibition of osteoclastogenesis while it could promote the osteogenic differentiation leading to a rapid regeneration of new bone and reunion of the bone marrow cavity. Ge and coworkers [83] developed a small intestinal submucosa (SIS) modified polypropylene (PP) scaffold via the PDA-coated technology for pelvic reconstruction. The* in vivo* rat model study showed that SIS attached to PP via PDA indicated a low expression of proinflammatory but a high expression of promacrophages which significantly contributed to pelvic tissue reconstruction. Study conducted by Madhurakkat Perikamana and coworkers [84] used a poly(L-lactic acid) (PLLA) nanofibril scaffold with an aligned morphology to explore the* in vivo* collagen assembly of regenerated bone via a mouse calvarial defect model. They utilized PDA-coated technology to immobilize bone morphogenic protein-2 (BMP-2) on the aligned PLLA nanofibers. The surface immobilized BMP-2 via PDA coating successfully controlled collagen fiber assembly in the* in vitro* environmental, which may be promising to be used to engineer structurally relevant bone. This scaffold also played an active role in the new bone formation where the* in vivo* bone regeneration was significantly better than control groups without PDA binding BMP-2. Li and coworkers [85] also exploited PDA-coated technology to immobilize bone forming peptide-1 (BFP-1) onto the poly(lactic-co-glycolic acid) (PLGA) surface. BFP-1 modified PLGA scaffold achieved a higher osteogenic activity than BMP-2 and the* in vivo* study further demonstrated that it significantly promoted bone formation in a nude murine model. Gao and coworkers [86] used PDA-coated technology to bind nanohydroxyapatite (nano-HA) rather than bone growth factors onto the polycaprolactone (PCL) surface. The results showed that nano-HA modified PCL via PDA coating enhanced the cell adhesion and proliferation, as well as promoting the bone regeneration even in the absence of osteogenesis soluble inducing factors in* in vivo* tests. In all the above examples, the use of PDA-coated technology is a relatively simple and rapid process requiring only a material-immersing preparation. Various active molecules can be immobilized on various surfaces via such a fascinating PDA-coating chemistry, which thereby greatly offers considerable merits to bone regeneration.

## 5. Material Categories of Bone Biosubstitutes Adapted to PDA-Assisted Modification

Most applications of PDA-assisted surface modification are used in bone tissue engineering. As a qualified bone biosubstitute, it should offer good biocompatibility, multiple functions, and other superior physical properties. Compared to traditional surface modification approaches including physical deposition/absorption, chemical modification/grafting, and plasma technology, the PDA-assisted surface modification may remedy some drawbacks from traditional approaches [71]. Different material categories of bone biosubstitutes must be adapted to PDA-assisted modification and the processing of PDA coating layer may also vary with different material categories to achieve the best outcome. Two kinds of typical materials that are used to prepare bone biosubstitutes can achieve a significant improvement of surface properties via PDA-assisted surface modification, which would be conducive to the cell growth and tissue regeneration.

Metallic materials and their alloys are one important category of materials used to fabricate bone biosubstitutes for many orthopedic operations, such as bone fracture and articular replacement. For orthopedic and dental implants, Ti6Al4V is a broadly used metallic composite material consisting of inorganic materials, metallic titanium, and its alloy. Once it is coated by the PDA layer, a special strong charge transfer interaction would be formed between the formed PDA coating layer and Ti, which can offer the biosubstitute a strong corrosive resistance, an improved mechanical properties, and a high superficial energy [87]. In addition, the PDA-assisted surface modification to Ti and Ti alloys can also offer the osteoinductivity and osteoconductivity to bioinert metallic bone biosubstitutes and enhance their bioactivities regarding the viability, proliferation, and differentiation of cell and material mineralization [23, 71, 72, 88]. Furthermore, the PDA coating layer can effectively immobilize vascular endothelial growth factor (VEGF) on the Ti-based biosubstitute without the adverse effect on the activity of VEGF [89]. Such a layer-by-layered composite bone biosubstitute combining VEGF, PDA layer, and Ti material significantly facilitates the attachment and proliferation of endothelial cells, as well as benefiting the differentiation of human mesenchymal stem cells (hMSC) to endothelial cells. In light of various functions obtained by immobilizing different biomolecules, endeavors related to the PDA-assisted surface modification on diverse metallic surfaces are being or have been performed to introduce new concepts and new processing strategies in order to enhance the revascularization ability, haemocompatibility, and antibacterial ability during the period of bone regeneration [89].

Synthetic and bioderived polymers are another important category of materials used to fabricate bone biosubstitutes, such as poly(l-lactide-co-glycolide) (PLGA), polycaprolactone (PCL), PLLA, collagen, and cellulose. Although most polymeric biomaterials are biodegradable, biocompatible, and erosion-resistant and have appropriate mechanical properties, they lack satisfactory osteointegration [90]. Through the PDA-assisted surface modification, the surface properties of these polymers can be greatly improved. Upon immobilization of BMP, the property of osteoblast cells responsive to polymeric materials can be remarkably improved, and the material mineralization can be expedited in the presence of immobilized growth factors [91–95]. Bone filling materials are broadly used in bone tissue engineering, such as plastic surgery and bone defect. The PDA coating layer on poly(dimethylsiloxane) (PDMS) can enhance the cytoaffinity of PDMS based bone cement where NIH3T3 fibroblast cells can attach more readily to the material substrate which further improves the cell proliferation [96].

## 6. Conclusions and Prospectives

In order to construct a multifunctional bone biosubstitute, the surface of the biosubstitute is important to combine stable functional factors and provide satisfactory cell behavior containing cell attachment, proliferation, migration/spreading, and differentiation to facilitate the bone tissue regeneration. PDA-assisted surface modification uses PDA as a biocompatible surface modifier to interact with organic and inorganic components on the interface of bone bio-substitutes. Such targeted modification makes the biphasic interface of material more stable and versatile for diverse requirements from bone tissue repair [97]. Two key advantages of PDA-assisted surface modification of bone biosubstitutes are as follows: (1) the PDA coating layer can be formed under a mild alkaline condition only with the contact of oxygen to obtain relatively satisfactory properties and other methods containing enzymatic oxidation and electropolymerization may form PDA with higher quality in a benign environment or in shorter reaction time [98]; (2) the autopolymerization of PDA precursor (DA) can occur virtually on various surfaces of materials, such as noble metals, oxide, polymers, and ceramics. As compared to traditional approaches with regard to surface modification or biomolecule immobilization, the PDA-assisted surface modification exhibits some exceptional characters containing facile processing, stable performance, and versatile application. Although the mechanism behind the formation of PDA coating layer on the material substitute still remains under debate, the PDA-assisted surface modification is expanding its applications to not only bone biosubstitutes but also cardiovascular devices, biosensors, neuron pseudosubstitutes, and sustainable drug delivery [18, 99–101].
